# Development and external validation of a prediction model for 90-day readmission in elderly patients with COPD complicated by pulmonary heart disease

**DOI:** 10.3389/fmed.2026.1830474

**Published:** 2026-06-11

**Authors:** Guibin Zhang, Dan Wang, Hang Chen, Wenchao Dai, Xingfu Fan, Yulian Liu, Juan Du, Li Jiang

**Affiliations:** 1 Department of Respiratory and Critical Care Medicine, Affiliated Hospital of North Sichuan Medical College, Nanchong, Sichuan, China; 2 Department of General Medicine, Affiliated Hospital of North Sichuan Medical College, Nanchong, Sichuan, China; 3 Department of Respiratory and Critical Care Medicine, Dazhou Hospital of Integrated Traditional and Western Medicine, Dazhou, Sichuan, China; 4 Department of Respiratory and Critical Care Medicine, People's Hospital of Guang'an District, Guang'an, Sichuan, China

**Keywords:** 90-day readmission, COPD, external validation, pulmonary heart disease, Shap

## Abstract

**Background:**

Older individuals developing chronic obstructive pulmonary disease (COPD) and concurrent pulmonary heart disease (PHD) have a higher early readmission probability post-discharge. But validated and interpretable models for forecasting 90-day COPD/PHD-related readmission among such individuals remain scarce.

**Methods:**

This retrospective two-center study recruited ≥ 65-year-old patients developing COPD and concurrent PHD from Affiliated Hospital of North Sichuan Medical College (development cohort) as well as Hospital of Integrated Traditional Chinese and Western Medicine of Dazhou City (external validation cohort). Candidate predictors were selected from pre-discharge routine data during index hospitalization, encompassing demographics, comorbidities, laboratory tests during the first 24 h post-admission, primary transthoracic echocardiographic parameters obtained during index hospitalization, and in-hospital non-invasive ventilation (NIV) use. We excluded troponin T and procalcitonin out of analysis because of massive missingness. We utilized multiple imputation for processing other missing data via chained equations. LASSO logistic regression was carried out for predictor selection by 10-fold cross-validation. Five models, consisting of logistic regression (LR), XGBoost, linear support vector machine, naive Bayes, and decision tree, were constructed, followed by performance comparison. Following calibration of model probabilities, the threshold acquired based on the development cohort was applied in internal bootstrap and external validation. Additionally, model performance was evaluated by calculating area under the receiver operating characteristic (ROC) curve (AUC), accuracy, sensitivity, specificity, as well as Brier score. At last, SHAP analysis and nomogram development were used for the final model.

**Results:**

There were altogether 588 patients enrolled, involving 433 and 155 in the development and external validation cohorts separately. XGBoost had the best apparent performance in the development cohort, however, LR showed the highest external discrimination (AUC 0.825) and stable calibration for 90-day COPD/PHD-related readmission. The SHAP analysis-identified predictors were TRVmax, frequent exacerbations, and NIV use.

**Conclusion:**

LR demonstrates the highest robustness and generalizability in terms of its performance, meanwhile, it maintains its clinical interpretability and transparency. Therefore, it can be applied in discharge planning for 90-day COPD/PHD-related readmission among old COPD patients with PHD.

## Introduction

As population aging accelerates, chronic respiratory diseases contribute increasingly to disease incidence and healthcare utilization among the elderly. Chronic obstructive pulmonary disease (COPD), which shows the typical features of continuous respiratory symptoms, chronic airway inflammation, and airflow limitation, shows the highest prevalence in these people (1). According to the national survey carried out during 2014–2015 in China, the age-standardized COPD prevalence in people ≥ 40 year was 13.6%, and such rate remarkably increased with age (2).

In the course of COPD, acute exacerbations contribute disproportionately to symptom burden and healthcare use^3 4^. Acute exacerbation of COPD (AECOPD), which is conventionally defined as an acute, sustained worsening of respiratory symptoms beyond usual day-to-day variation in a previously stable patient, prompts additional treatment or adjustment of maintenance therapy (3). These exacerbations are typically accompanied by pronounced symptom worsening and physiological instability, and are consistently associated with increased morbidity and mortality (4). Besides, they tend to recur frequently. As revealed by prospective cohort studies, a greater number of frequent exacerbations (FE) relate to decreased lung function, and the forced expiratory volume in 1 s (FEV_1_) decreases substantially in “frequent exacerbators” (5). For an exacerbation requiring hospitalization, the prognostic outcome is extremely dismal, and a higher age can independently predict unfavorable results (6, 7). Recovery is often slow; for some patients, symptoms and lung function remain below baseline levels for weeks, a window during which recurrence and readmission are more likely to occur (8). These observations underscore the importance of tailored post-discharge management in older patients with AECOPD to reduce short- to medium-term readmissions^6 8^.

Pulmonary heart disease (PHD) is a clinically significant cardiovascular comorbidity in older patients hospitalized with AECOPD. It is usually caused by chronic hypoxaemia with secondary pulmonary hypertension (PH), which imposes a sustained increase in right ventricular afterload. Cardiopulmonary reserve is usually restricted during an exacerbation, accompanied by insufficient compensatory responses, which may worsen the episode and delay post-discharge recovery (9). Fluctuations of pulmonary artery pressure are recently reported in AECOPD, and greater pressures are related to more dismal prognosis. Such results provide the mechanistic foundation for the elevated early readmission risk among old patients who develop PHD or PH (9). Transthoracic echocardiography, a widely available tool in routine practice, enables non-invasive assessment of right heart involvement and PH risk. Tricuspid regurgitation velocity (TRV), a frequently utilized metric, can be used to evaluate pre-discharge risk and integrate in prediction models (10).

Research regarding COPD exacerbations and readmission is mostly carried out with different cohorts, and modeling process and outcome definitions vary considerably among studies. Just few studies conduct external validation, but their methodological quality is heterogeneous. The above-mentioned limitations greatly hinders clinical translation (11). There are clinical risk scores proposed for patients hospitalized with AECOPD. For example, the Previous admissions, Extended Medical Research Council dyspnoea score, Age, Right-sided heart failure, and Left-sided heart failure (PEARL) score was developed to predict the risk of 90-day readmission or mortality (12). In parallel, machine learning models using electronic health record data have also been trained to predict 90-day readmission in this setting (13). Nonetheless, key questions persist. It remains unclear whether these models maintain their performance across hospitals with different clinical practices and patient populations. In addition, they have uncertain interpretability at the bedside. Collectively, these issues are still the major obstacles to broader model implementation.

Accordingly, this study focused on a high-risk subgroup: older patients with COPD complicated by PHD. The primary outcome was COPD/PHD-related readmission within 90 days after discharge. Routinely collected data from early hospitalization and the pre-discharge period, including clinical characteristics, laboratory test results, and the first in-hospital echocardiographic assessment, were utilized. On the basis of the above variables, we constructed multiple prediction models and assessed their performance in the independent external cohort. To increase clinical usefulness, we also used simple risk visualization tools and conducted feature attribution analyses. Our findings in the present work will assist in personal risk assessment for discharge planning and post-discharge care.

## Methods

### Ethics statement

The Ethics Committee of Affiliated Hospital of North Sichuan Medical College approved this study (Approval No.: 2025ER107-1). Dazhou Hospital of Integrated Traditional and Western Medicine, the sub-center, verified that the protocol merely involved secondary analyses on available, de-identified clinical information. Further ethics review was waived following local governance requirements and institutional policy, with no separate application submitted. Patient records were anonymized before analysis. This study was carried out strictly following the Declaration of Helsinki as well as applicable local laws and regulations.

### Study population

The current retrospective two-center study recruited ≥ 65-year-old COPD inpatients with PHD from Department of Respiratory and Critical Care Medicine of Affiliated Hospital of North Sichuan Medical College from July 2022 to January 2025, and from Dazhou Hospital of Integrated Traditional and Western Medicine from January to July 2025. The diagnosis of COPD was made according to post-bronchodilator spirometry exhibiting a FEV_1_/FVC ratio < 0.70, or a prior diagnosis reported in the medical record (14). To assess PHD, transthoracic echocardiography and/or electrocardiography (ECG) was performed. Typically, PHD was diagnosed according to the criteria below: (1) P pulmonale on ECG; (2) right ventricular hypertrophy on ECG; (3) PH on echocardiography; and (4) right ventricular hypertrophy or dilatation on echocardiography (15).

Patients below were eliminated: (1) concurrent asthma, malignancies or active pulmonary tuberculosis; (2) in-hospital mortality; (3) autoimmune disorder; (4) additional acute conditions (like acute myocardial infarction); (5) severe psychological/psychiatric conditions potentially compromising data quality or result evaluation; and (6) unavailable primary outcome data or key covariates.

### Outcome definition and ascertainment

The primary outcome was COPD/PHD-related readmission within 90 days after discharge from the index hospitalization. Readmission was defined as an unplanned inpatient admission primarily attributed to AECOPD and/or PHD-related decompensation, based on admission notes and discharge diagnoses. Outcomes were identified in each participating hospital using local outpatient and inpatient systems, including the electronic medical record, clinic visit records, and admission/discharge databases. Due to the lack of regional data linkage, claims access and standardized telephone follow-up, admissions to other hospitals could not be tracked. As a result, readmissions occurring outside the participating hospitals may have been missed.

### Data extraction and processing

Demographics (age, sex, body mass index [BMI], smoking history), comorbidities (hypertension, diabetes, cardiovascular disease), and in-hospital non-invasive ventilation (NIV) use were collected. Laboratory parameters encompassed platelet count, white blood cell count with differential, hemoglobin, red blood cell distribution width (RDW), D-dimer, coagulation indexes, alongside routine biochemistry (albumin [ALB], creatinine). In the meantime, echocardiographic variables were ejection fraction (EF), right atrial end-systolic dimensions, right ventricular diameter, peak tricuspid regurgitation velocity (TRVmax), main pulmonary artery diameter, as well as pulmonary artery systolic pressure. Additionally, the FE (≥2 hospitalizations due to acute exacerbation within the preceding year) history was obtained. Only variables documented during the index admission and accessible prior to discharge were considered candidate predictors. The outcome was assessed over a 90-day period after discharge. For laboratory tests, the first set of results obtained within 24 h of admission were used. NIV use was defined as any in-hospital application of NIV during the index admission prior to discharge, as documented in medical orders and nursing or respiratory therapy records. Echocardiographic variables were derived from the first examination performed during the hospital stay. If multiple records were available, the earliest measurement was used. Comorbidities and smoking status were obtained from medical history and discharge diagnoses and verified against supporting examinations where applicable.

Missing data were handled separately in the development and external validation cohorts to account for center-specific data patterns. Variables with more than 30% missing values were excluded from analysis. The remaining missing values were imputed using multiple imputation by chained equations (MICE). Predictive mean matching was employed for continuous variables, logistic regression for binary variables, whereas multinomial logistic regression for categorical variables with more than two levels. All analyses were conducted across 20 imputed datasets, and results were combined using Rubin’s rules (16).

### Statistical analysis

R software (version 4.5.1) was employed for statistical analysis. Baseline data in both cohorts were obtained, respectively. Continuous data were represented by mean ± standard deviation, whereas categorical counterparts by number (percentage). This baseline table was used for describing between-cohort case-mix differences rather than hypothesis testing, model-building decisions or feature selection.

We used the main-center cohort to develop a model and conduct internal validation, and the sub-center cohort for external validation. The primary outcome was 90-day readmission after discharge. Prior to modeling, categorical variables were converted to factors and harmonized across cohorts, and binary variables (e.g., FE and NIV) were coded as “No/Yes.” Model development was completed following a prespecified pipeline. Candidate predictors were initially screened using least absolute shrinkage and selection operator (LASSO) logistic regression with 10-fold cross-validation, with lambda.min being selected to determine the final feature set (17). To ensure comparability, all subsequent models were trained using the same selected features. Five models were then fitted: logistic regression (LR), eXtreme Gradient Boosting (XGBoost), support vector machine (SVM), naïve Bayes (NB), and decision tree (DT). For the machine-learning models, the main hyperparameters were set before model training, without an additional grid-search procedure. XGBoost was fitted with a binary logistic objective and AUC-based evaluation, using conservative settings for tree complexity and subsampling. The SVM model used a linear kernel with probability estimation, whereas the decision tree and naïve Bayes models were fitted with package-default settings. Class imbalance was not handled by synthetic oversampling or undersampling methods such as SMOTE. Instead, we used AUC-based discrimination assessment, cross-validated out-of-fold predictions, Platt calibration, and outcome-stratified bootstrap resampling. In the bootstrap procedure, patients with and without 90-day readmission were resampled separately with replacement to preserve the event/non-event distribution.

In the development cohort, 10-fold cross-validation was employed to generate out-of-fold (OOF) predicted probabilities for each model (17). These OOF probabilities were later calibrated using Platt scaling, implemented as logistic regression of the observed outcome on the logit-transformed OOF probabilities [i.e., pcal = logistic (a + b × logit(praw))]. The Youden index was used to determine the operating threshold for calibrated OOF predictions. Then, this threshold was also used in subsequent assessments to maximally reduce validation bias while enhancing reproducibility.

The area under the receiver operating characteristic (ROC) curve (AUC) values were calculated for assessing model discrimination. Performance was evaluated by accuracy, sensitivity and specificity at the predetermined threshold, while ROC curve analysis was carried out for evaluating discrimination. Calibration curves and Brier score were adopted to evaluate calibration, while decision curve analysis (DCA) was conducted to assess clinical utility. Internal validation was carried out using 500 outcome-stratified bootstrap resamples (18), and the full analytical workflow, including feature selection, model fitting, calibration and evaluation, was repeated in each resample to derive optimism-corrected performance estimates. Eventually, external validation was conducted in the independent sub-center cohort (19). To enhance model interpretability, SHapley Additive exPlanations (SHAP) analysis, implemented using the fastshap and shapviz packages, was applied to the final selected model in the development cohort to quantify the direction and magnitude of feature contributions.

To facilitate independent replication and clinical implementation beyond the graphical nomogram, the complete final LR specification is provided in Supplementary Table S1-S2, covering the intercept and all regression coefficients, variable coding (with reference levels and dummy-variable encoding), measurement units, Platt calibration parameters (a, b), and the prespecified operating threshold.

## Results

### Baseline characteristics

Troponin T (TnT) and procalcitonin (PCT) were excluded from further analyses due to more than 30% missing data, as a result, 37 candidate variables were retained for subsequent analyses. There were altogether 433 and 155 cases enrolled into the development and external validation cohorts. Both cohorts display similar case-mix features: males occupied about 2/3 of patients (68.4% vs. 67.1%), smokers took up 1/2 (50.6% vs. 51.6%), and NIV use rates were 60.0 and 59.4% separately. Besides, both cohorts showed a similar FE history (35.8% vs. 39.4%). Comorbidities, such as cardiovascular disease (51.5% vs. 47.7%), hypertension (32.6% vs. 34.8%), and diabetes (16.9% vs. 18.1%), were similar between cohorts. The sub-center cohort had a higher 90-day readmission rate (29.7% vs. 20.6%). The mean age was 76.2 and 76.8 years in both cohorts, whereas the BMI was 21.3 and 21.8 kg/m^2^, separately. Arterial blood gas parameters, ECG variables and routine laboratory parameters were similar in both cohorts (Table 1). To further characterize outcome-related baseline differences within the development cohort, we compared baseline characteristics between patients with and without 90-day COPD/PHD-related readmission. The detailed results are presented in Supplementary Table S3.

**Table 1:** Baseline characteristics comparison between the main-center (development) and sub-center (external validation) cohorts.

Characteristics	Main center	Sub center
	*N* = 433	*N* = 155
Age (year)	76.2 (6.8)	76.8 (5.2)
Gender		
Female	137 (31.6%)	51 (32.9%)
Male	296 (68.4%)	104 (67.1%)
BMI (kg/m^2^)	21.3 (3.6)	21.8 (3.1)
FE		
No	278 (64.2%)	94 (60.6%)
Yes	155 (35.8%)	61 (39.4%)
Smoke		
No	214 (49.4%)	75 (48.4%)
Yes	219 (50.6%)	80 (51.6%)
NIV		
No	173 (40.0%)	63 (40.6%)
Yes	260 (60.0%)	92 (59.4%)
RF		
No	201 (46.4%)	72 (46.5%)
Yes	232 (53.6%)	83 (53.5%)
Readmission		
No	344 (79.4%)	109 (70.3%)
Yes	89 (20.6%)	46 (29.7%)
Hypertension		
No	292 (67.4%)	101 (65.2%)
Yes	141 (32.6%)	54 (34.8%)
CVD		
No	210 (48.5%)	81 (52.3%)
Yes	223 (51.5%)	74 (47.7%)
Diabetes		
No	360 (83.1%)	127 (81.9%)
Yes	73 (16.9%)	28 (18.1%)
PaO_2_ (mmHg)	85.1 (29.2)	87.2 (36.3)
PaCO_2_ (mmHg)	52.4 (17.3)	51.6 (14.1)
D (Ug/ml)	2.7 (4.5)	2.8 (3.2)
TT (s)	18.4 (1.3)	18.4 (1.3)
Hb (g/L)	129.3 (25.3)	128.3 (24.0)
APTT (s)	36.8 (9.3)	37.0 (10.3)
PTA (%)	81.7 (25.0)	80.6 (16.0)
BNP (pg/ml)	3172.3 (5285.9)	3237.3 (3102.5)
ALB (g/L)	37.3 (4.4)	37.2 (4.9)
Creatinine (umol/L)	78.7 (35.1)	85.8 (36.6)
WBC (10E9/L)	7.9 (3.6)	8.0 (3.7)
RBC (10E12/L)	4.4 (0.8)	4.3 (0.7)
HCT (%)	0.4 (0.1)	0.4 (0.1)
RDW (%)	14.8 (2.0)	14.7 (1.5)
PLT (10E9/L)	184.5 (82.2)	180.4 (69.8)
Neutrophil (10E9/L)	6.3 (3.4)	6.5 (3.5)
MPV (fl)	10.5 (1.6)	10.5 (2.1)
Lymphocyte (10E9/L)	1.0 (0.6)	1.1 (0.6)
Monocyte (10E9/L)	0.5 (0.3)	0.5 (0.3)
Eosinophils (10E9/L)	0.1 (0.1)	0.1 (0.1)
Basophil (10E9/L)	0.0 (0.0)	0.0 (0.0)
RVEDD (mm)	26.5 (5.1)	27.2 (5.6)
RALD (mm)	47.3 (8.4)	47.1 (10.3)
MPAD (mm)	25.3 (4.4)	24.9 (6.2)
EF (%)	61.4 (8.3)	60.1 (10.5)
TRVmax (m/s)	3.5 (0.6)	3.6 (0.6)
PASP (mmHg)	56.0 (14.4)	58.0 (15.7)

FE, frequent exacerbations (≥2 hospitalizations for acute exacerbations in the previous year); NIV, non-invasive ventilation; RF, Respiratory failure; CVD, Cardiovascular disease(s); D, D-dimer; TT, thrombin time; APTT, activated partial thromboplastin time; PTA, prothrombin activity; BNP, B-type natriuretic peptide; ALB, albumin; WBC, white blood cell count; RBC, red blood cell count; HCT, hematocrit; RDW, red cell distribution width; PLT, platelet count; MPV, mean platelet volume; RVEDD, right ventricular end-diastolic diameter; RALD, right atrial longitudinal diameter; MPAD, main pulmonary artery diameter; EF, ejection fraction; TRVmax, maximum tricuspid regurgitation velocity; PASP, pulmonary artery systolic pressure.

### Variable screening

**Figure 1:**
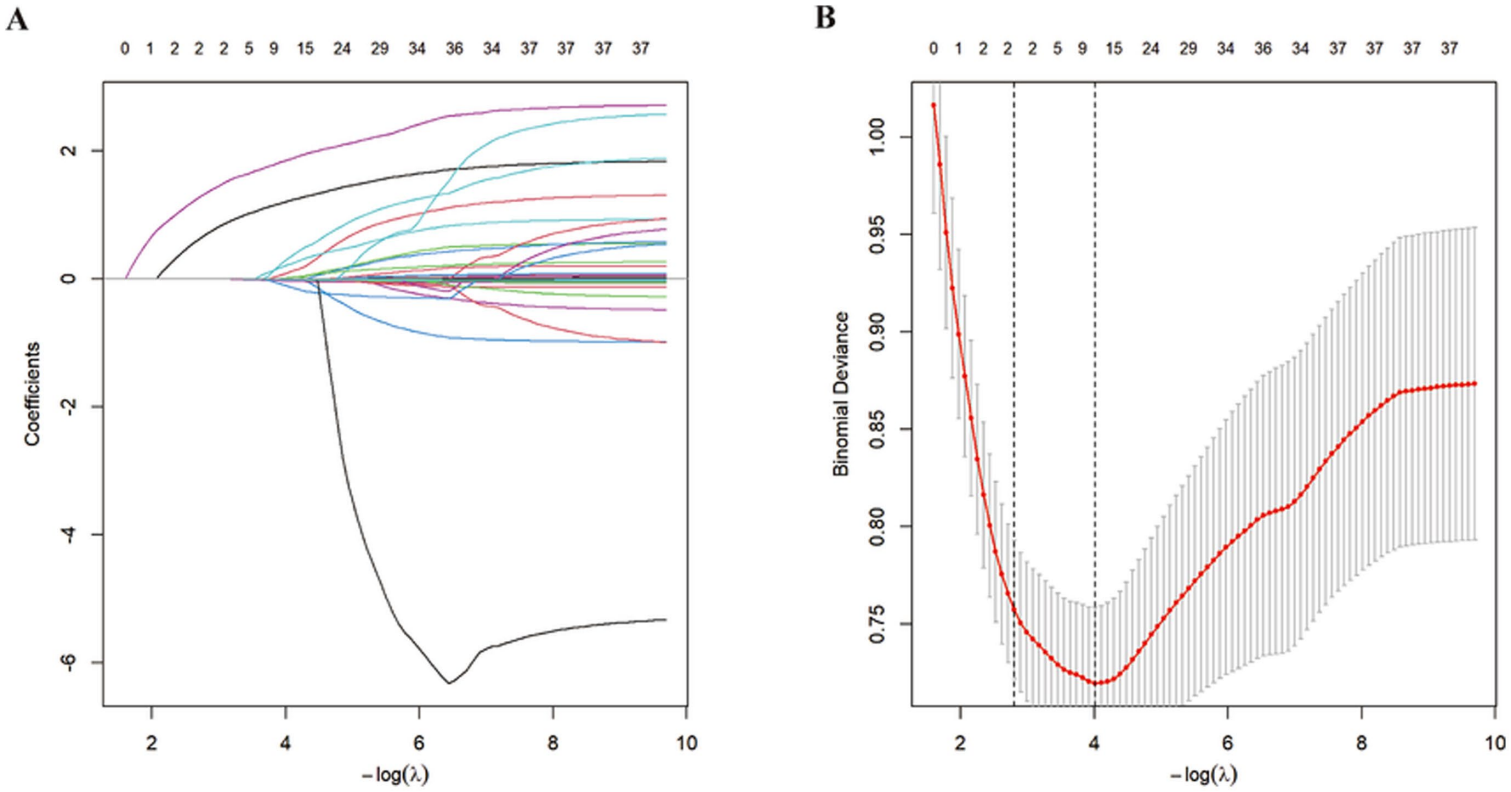
LASSO logistic regression coefficient profiles and cross-validation. **(A)** Coefficient paths as a function of −log(); each colored curve represents one predictor. Numbers along the top indicate the number of non-zero coefficients (selected predictors) at each value of *λ*. **(B)** Cross-validated binomial deviance versus −log(λ). The red curve shows the mean deviance and gray bars indicate ±1 standard error. The two dashed vertical lines denote λ_min (minimum mean deviance) and 2_1se (largest a within 1 standard error of the minimum), respectively.

To address collinearity and limit overfitting, LASSO logistic regression with 10-fold cross-validation was utilized for predictor selection in the development cohort (Figure 1). As the penalty parameter (*λ*) increased, coefficients were progressively shrunk toward zero (Figure 1A). Then, λ_min, defined as the value giving the lowest cross-validated binomial deviance, was selected (Figure 1B). Variables with non-zero coefficients at λ_min were retained, resulting in 10 predictors: TRVmax, FE (defined as ≥ 2 AECOPD-related hospitalizations in the preceding year), NIV use, monocyte count, activated partial thromboplastin time (APTT), ALB, EF, lymphocyte count, sex, and mean platelet volume (MPV). These variables were used in subsequent model development and validation.

### Model performance comparison

**Figure 2:**
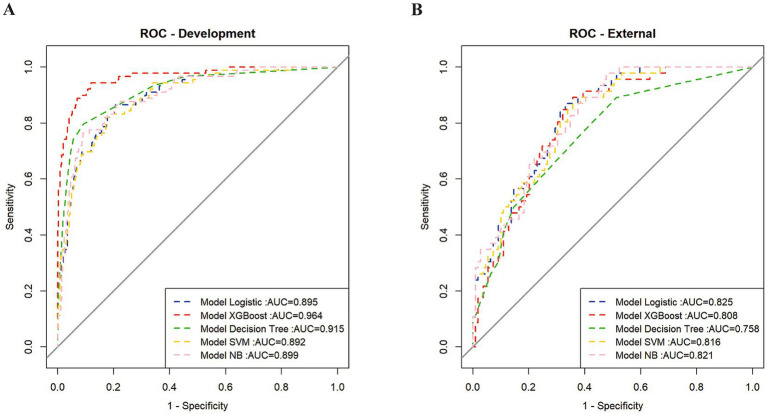
Receiver operating characteristic (ROC) curves for model discrimination in the development and external validation cohorts. **(A)** ROC curves in the development cohort comparing logistic regression (AUC = 0.895), XGBoost (AUC = 0.964), decision tree (AUC = 0.915), support vector machine (AUC = 0.892), and naïve Bayes (AUC = 0.899). **(B)** ROC curves in the external cohort comparing logistic regression (AUC = 0.825), XGBoost (AUC = 0.808), decision tree (AUC = 0.758), support vector machine (AUC = 0.816), and naïve Bayes (AUC = 0.821). The gray diagonal line indicates no-discrimination performance (AUC = 0.5).

Figure 2 and Table 2 display model performance in both cohorts. XGBoost showed the best discrimination in the main-center cohort (AUC = 0.964), then DT (AUC = 0.915) and LR (AUC = 0.895). With regard to SVM and NB, their AUCs were 0.892 and 0.899 separately (Figure 2A). To assess possible overfitting, 500 bootstrap resamples were used with optimism correction. The apparent AUC for LR was 0.895, with the mean optimism and optimism-corrected AUC of 0.054 and 0.841. The corrected AUCs for XGBoost, DT, SVM and NB were 0.913, 0.851, 0.842, and 0.851 separately. Such results demonstrate optimism to a certain degree in development cohort, but discrimination was still acceptable post-correction.

**Table 2:** Performance of four models in Development and External cohorts.

Model	AUC	Accuracy	Sensitivity	Specificity	Brier.score
Development					
LR	0.895	0.801	0.865	0.785	0.098
XGB	0.964	0.880	0.944	0.863	0.062
DT	0.915	0.885	0.798	0.907	0.089
SVM	0.892	0.811	0.831	0.805	0.098
NB	0.899	0.880	0.764	0.910	0.096
External					
LR	0.825	0.723	0.674	0.743	0.162
XGB	0.808	0.742	0.652	0.780	0.176
DT	0.758	0.748	0.500	0.853	0.180
SVM	0.816	0.716	0.696	0.725	0.163
NB	0.821	0.723	0.500	0.817	0.157

AUC, Area Under the Curve; LR, Logistic Regression; XGB, eXtreme Gradient Boosting; DT, Decision Tree; SVM, Support Vector Machine; NB, naive Bayes.

**Figure 3:**
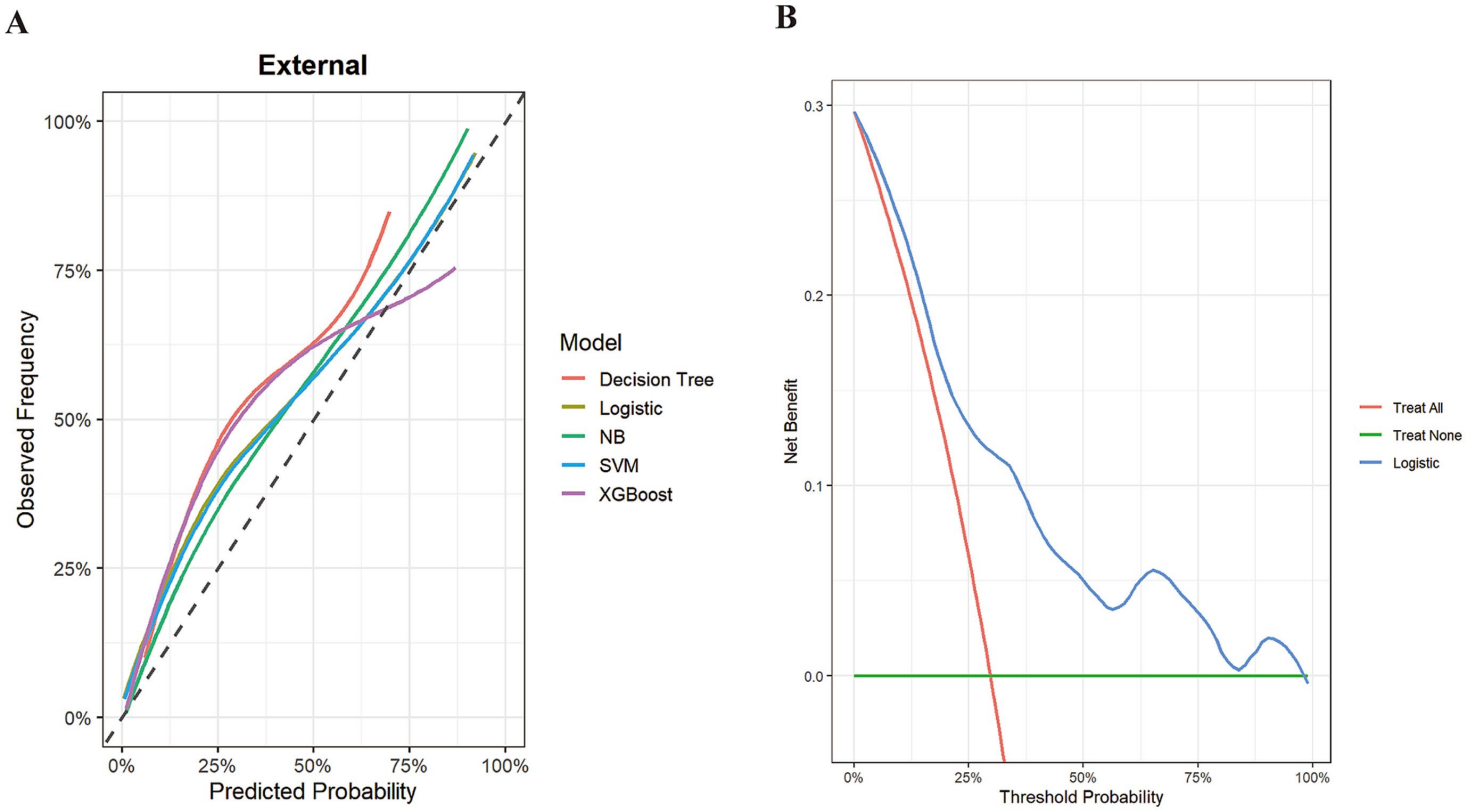
Model calibration and decision curve analysis in the external validation cohort. **(A)** Calibration plots showing agreement between predicted probabilities and observed event frequencies for the decision tree, logistic regression, naïve Bayes (NB), support vector machine (SVM), and XGBoost models. The dashed diagonal line represents perfect calibration. **(B)** Decision curve analysis (DCA) comparing the net benefit of the logistic regression model (blue) with the default strategies of treating all patients (red) and treating none (green) across a range of threshold probabilities.

In the external validation cohort, models were ranked by discriminative ability as follows: LR achieved the highest AUC (0.825), followed by NB (AUC = 0.821), SVM (AUC = 0.816), and XGBoost (AUC = 0.808), whereas DT exhibited the lowest discriminative performance (AUC = 0.758; Figure 2B). Using the threshold predefined in the development cohort based on calibrated OOF predictions, LR achieved an accuracy of 0.723, a sensitivity of 0.674, and a specificity of 0.743 in the external cohort, with a Brier score of 0.162 (Table 2). The calibration curve for LR also aligned more closely with the 45° reference line across most ranges of predicted risk (Figure 3A). DCA suggested a higher net benefit over a broader range of threshold probabilities than the “treat-all” and “treat-none” strategies (Figure 3B). Considering external discriminative ability, robustness in internal validation, calibration, and clinical interpretability, LR was selected as the final model for subsequent interpretation and potential clinical application.

### Model interpretation

**Figure 4:**
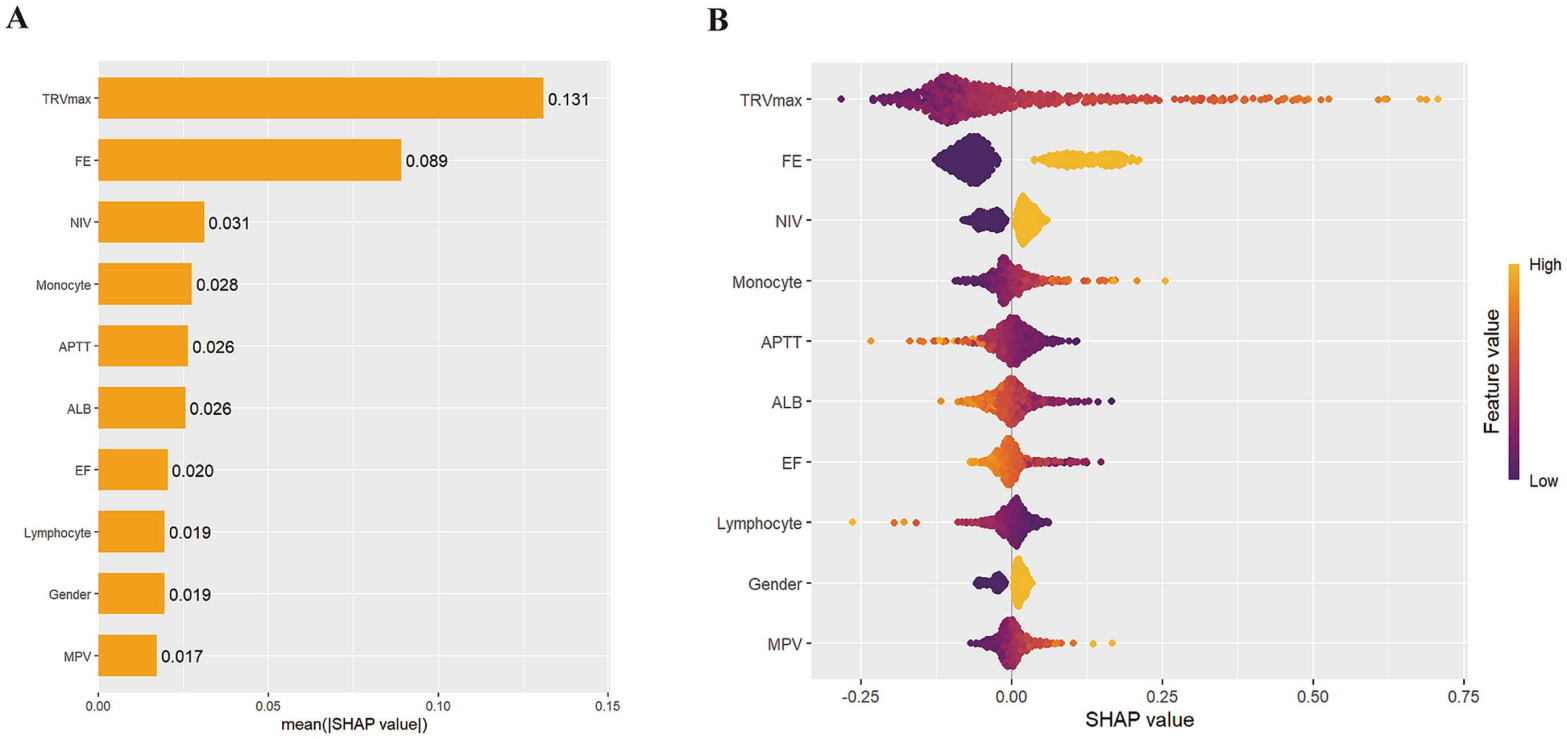
SHAP-based interpretation of the final model. **(A)** Global feature importance ranked by the mean absolute SHAP value. **(B)** SHAP summary (beeswarm) plot showing the distribution of SHAP values for each feature across individuals; point color denotes the corresponding feature value (low to high), where positive SHAP values increase and negative values decrease the predicted risk. Variables: TRVmax, maximum tricuspid regurgitation velocity; FE, frequent exacerbation history (≥2 hospitalizations due to acute exacerbations within the past year); NIV, non-invasive ventilation; Monocyte, monocyte count; APTT, activated partial thromboplastin time; ALB, serum albumin; EF, left ventricular ejection fraction; Lymphocyte, lymphocyte count; MPV, mean platelet volume.

**Figure 5:**
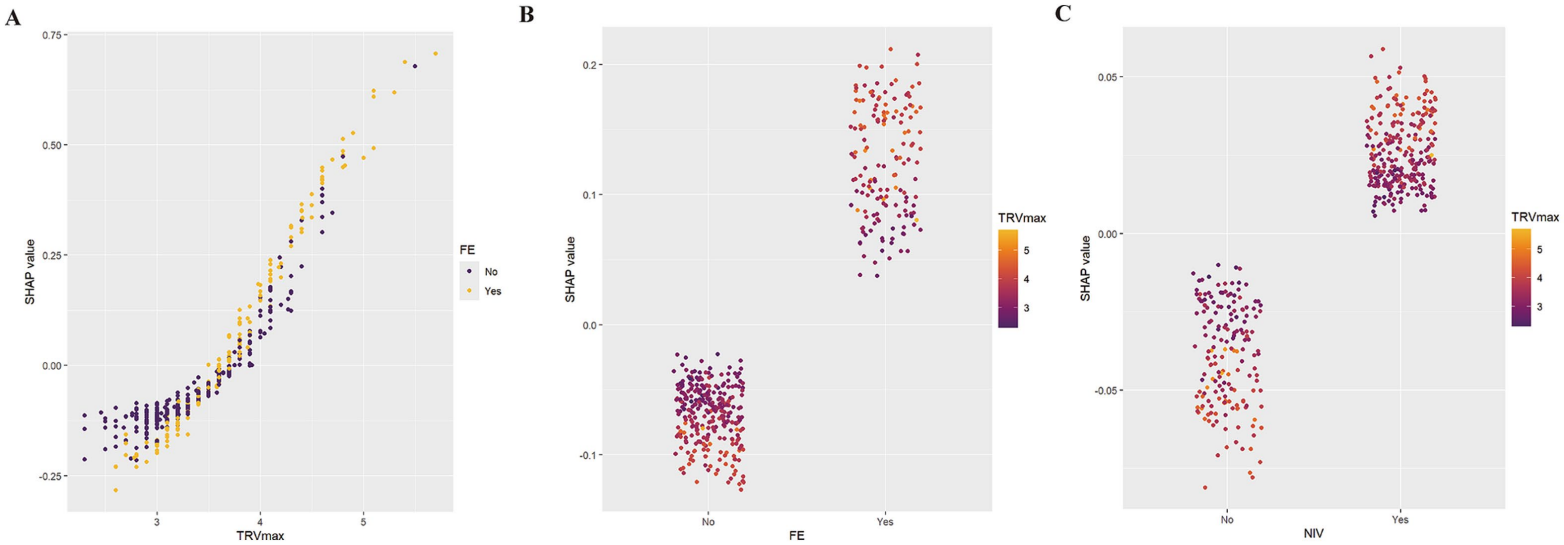
SHAP dependence plots illustrating the effects and interactions of key predictors on the model output. **(A)** Dependence of the SHAP value on TRVmax, with points colored by FE status (Yes/No), showing how the contribution of TRVmax varies with frequent exacerbation history. **(B)** SHAP values stratified by FE (Yes/No) and colored by TRVmax, indicating the joint influence of frequent exacerbation history and maximum tricuspid regurgitation velocity. **(C)** SHAP values stratified by NIV (Yes/No) and colored by TRVmax, demonstrating the interaction between non-invasive ventilation and maximum tricuspid regurgitation velocity on the predicted risk. Positive SHAP values indicate increased predicted risk, whereas negative values indicate decreased predicted risk. TRVmax, maximum tricuspid regurgitation velocity; FE, frequent exacerbation history (≥2 hospitalizations due to acute exacerbations within the past year); NIV, non-invasive ventilation; SHAP, SHapley Additive exPlanations.

We conducted SHAP analysis in the development cohort (Figures 4 and 5) to explore the influence of diverse variables on the predicted 90-day readmission probability, so as to improve the final LR model interpretability. TRVmax contributed most, followed by FE and NIV uses (mean |SHAP| = 0.131, 0.089 and 0.031 separately). Milder contributors were monocyte count, ALB, APTT, EF, lymphocyte count, sex, as well as MPV (Figure 4A). The direction of such effects was illustrated in the SHAP beeswarm plot. Notably, greater TRVmax, FE = Yes, and NIV = Yes predicted positive SHAP values, suggesting a higher readmission probability. Likewise, smaller ALB and EF indicated a higher readmission risk. The pattern is consistent with the increased risk seen in patients having poor nutritional status and declined cardiac function (Figure 4B).

From the dependence plot, TRVmax was positively related to SHAP values, and TRVmax increased with the growing SHAP values (Figure 5A). Besides, SHAP values were higher in those with FE = Yes relative to those with FE = No at each TRVmax level (Figure 5A). Subgroup analyses also indicated that SHAP values were mainly > 0 in FE = Yes group but < 0 in FE = No group. Similarly, the NIV = Yes group always had increased SHAP values compared with the NIV = No group (Figures 5B,C). From the above findings, predictors suggesting severe disease status are related to the increased 90-day readmission probability.

### Nomogram establishment

**Figure 6:**
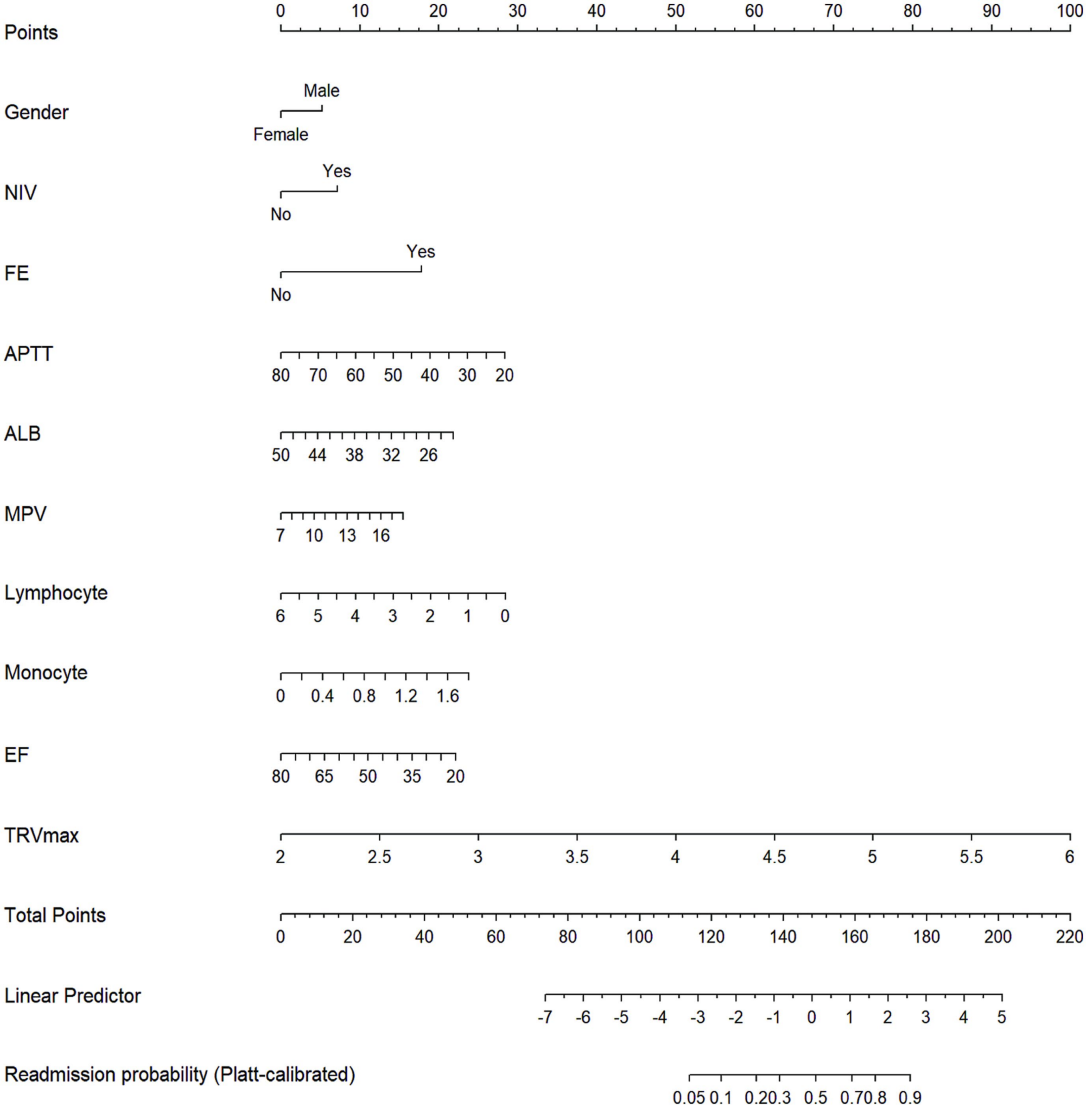
Platt-calibrated nomogram for predicting readmission risk based on the final logistic regression model. For each predictor (Gender, NIV, FE, APTT, ALB, MPV, Lymphocyte, Monocyte, EF, and TRVmax), locate the patient’s value on the corresponding axis and read the assigned score on the “Points” scale. Sum the individual scores to obtain the “Total Points,” then project vertically downward to derive the “Linear Predictor” and the Platt-calibrated probability of readmission. Higher total points indicate a higher predicted risk of readmission. NIV, non-invasive ventilation; FE, frequent exacerbations (22 hospitalizations for acute exacerbations in the previous year); APTT, activated partial thromboplastin time; ALB, albumin; MPV, mean platelet volume: EF, ejection fraction; TRVmax, maximum tricuspid regurgitation velocity.

To promote clinical use, we converted the Platt-calibrated LR model in a nomogram (Figure 6). This nomogram was developed by incorporating sex, NIV use, FE, APTT, MPV, ALB, lymphocyte count, monocyte count, EF, and TRVmax. All variables were read off the scales in each patient and assigned corresponding points. These points were later added up, and the total point was transformed into the “Readmission probability (Platt-calibrated)” for estimating 90-day readmission probability. This offers a useful strategy to use regression models to assess discharge risk and plan follow-up care.

The nomogram has been recognized as a useful tool, which must be utilized together with complete model specification. The complete final model is presented in Supplementary Tables S1 and S2. Supplementary Table S1 displays the intercept, coefficients, units, along with complete LR equation. Supplementary Table S2 shows variable coding and reference categories, the Platt calibration formula with parameters (a, b), as well as the operating threshold predetermined and used in the external validation cohort. The above tables help directly calculate calibrated probabilities without dependence on the nomogram.

## Discussion

Based on routine data in two institutions, we constructed the 90-day readmission prediction model for old COPD patients with PHD, and then validated it in the independent validation cohort. These models could distinguish between high- and low-risk patients in development cohort. However, different findings were obtained from the external cohort, revealing the influence of different patient populations and clinical practices among centers on model performance. Model stability was assessed with 500 bootstrap resamples and optimism correction. The apparent performance exhibited only modest optimism, and optimism-corrected estimates remained acceptable. LR was the most consistent method in external validation and was straightforward to interpret clinically, therefore, it was selected as the final model. Finally, SHAP analysis and calibrated nomogram development were performed to identify key predictors and illustrate their impact on the readmission risk, thus providing a practical framework for individualized readmission risk assessment and post-discharge planning.

Complex machine learning models often demonstrate superior discriminative ability in the development cohort due to their capacity to fit nonlinear effects and higher-order interactions. However, this advantage often diminishes when such models are tested in an independent external cohort (20, 21). In multi-center settings, site-to-site differences in case-mix, laboratory testing (frequency and assay methods), treatment strategies, and post-discharge care can shift the underlying data distribution, probably destabilizing more complex models when they are applied to a new population (21). In contrast, LR is more parsimonious and relies on fewer parameters, making it generally more tolerant to moderate changes in sample size and predictor distributions. Therefore, it may more likely retain stable performance during external validation in real-world clinical contexts (22, 23). In addition, feature selection, probability calibration, and the operating threshold were predefined in the development cohort, and the same threshold was consistently applied in external validation, thereby avoiding performance inflation caused by *post hoc* re-tuning in the validation cohort and enabling a fairer comparison across algorithms (24). Considering cross-center performance, bootstrap-based stability estimates, and the need for transparent clinical interpretation, LR was selected as the final model. The resultant model is more readily implemented in routine practice and can be operationalized as a simple bedside risk assessment tool.

In the interpretability analysis, TRVmax, a history of FE, and in-hospital NIV use contributed most to the predicted 90-day readmission risk. TRVmax was obtained from the first in-hospital transthoracic echocardiography and through the simplified Bernoulli relationship (ΔP ≈ 4 × TRVmax^2^). It reflects the tricuspid regurgitation pressure gradient and is commonly used as a bedside surrogate for pulmonary artery pressure and right heart load in the absence of right ventricular outflow obstruction. Moreover, it is also a key parameter for estimating the likelihood of PH (25, 26). In elderly COPD patients with concurrent PHD, a higher TRVmax likely indicates a heavier pulmonary vascular burden, an increased right ventricular afterload, and a reduced reserve—capturing both chronic hypoxia–related cardiopulmonary remodeling and the acute stress of an exacerbation (26). Previous studies have linked COPD-associated PH with a poorer clinical course and outcomes, and short-term increases in tricuspid regurgitation-related pressure during AECOPD have been associated with subsequent adverse events. These findings support the use of TRV-related measures as markers of early post-discharge vulnerability (9, 27, 28). Consistently, our SHAP dependence plot suggested a positive, nonlinear effect of TRVmax on model output, with a sharper rise in contribution at higher TRVmax values. Furthermore, larger contributions were observed among patients with a history of FE or NIV use, indicating an additive effect of right heart and pulmonary circulation strain and exacerbation severity. Overall, TRVmax measured on the first in-hospital echocardiography may help identify patients who require closer follow-up and more intensive cardiopulmonary-focused management after discharge.

Both FE (defined as ≥ 2 AECOPD-related hospitalizations in the preceding year) and early in-hospital NIV use made consistent contributions to our model, suggesting that they describe 90-day post-discharge vulnerability from two complementary perspectives: a tendency toward recurrent exacerbations and the severity of the index episode in the context of limited respiratory reserve (29, 30). Patients with a history of FE often align with a “frequent exacerbator” profile, in which prior events strongly predict future exacerbations and rehospitalization. Clinically, these patients may carry a higher inflammatory burden, have poorer lung function and oxygenation reserve, experience more comorbidities and frailty, and have greater difficulties in maintaining stable self-management after discharge, rendering them susceptible to a short-term cycle of exacerbation and readmission (29–31). For PHD patients, FEs combined with repeated hypoxemia and pulmonary vascular stress elevate pulmonary circulatory load while destabilizing right heart function, thus increasing the decompensation risk (32). Conversely, NIV use frequently suggests acute or progressive respiratory failure in admission, which usually shows the typical features of respiratory muscle fatigue, hypercapnia, or obvious ventilatory and gas-exchange derangement. For the elderly cor pulmonale patients, the limited reserve has almost no room for buffer post-discharge, and clinical deterioration (including progressive dyspnoea, cardiopulmonary decompensation and CO₂ retention) probably induces an increased rehospitalization risk (33). It is necessary to cautiously assess discharge risk and conduct post-discharge planning for patients reporting FEs and/or NIV use. This involves confirmation of stability/ventilation/oxygen targets pre-discharge, optimization of inhaled therapy, active management of triggers like infection, enhancement of pulmonary rehabilitation/comorbidity/frailty assessment, and arrangement of close early follow-up and interventions, so as to reduce the short-term readmission probability (31, 32).

In clinical use, calibration is as important as discrimination, since clinicians rely on absolute risk estimates rather than rank ordering alone. Therefore, Platt scaling was applied, and calibration and the Brier score were evaluated in the external validation cohort. Among the tested models, LR showed the closest agreement between predicted and observed risks in the external validation cohort. DCA further supported this choice: across a clinically plausible range of thresholds, the LR model yielded a greater net benefit than the “treat none” strategy, and became preferable to the “treat all” strategy once the threshold was around 30% or higher (Figure 3B). To facilitate clinical use and enhance interpretability, SHAP summaries for the final LR model were reported, including both overall and patient-level contributions (Figures 4 and 5), and a Platt-calibrated nomogram that enables point-based risk calculation at discharge was developed. Based on pre-discharge available data, clinicians are able to predict patients’ 90-day readmission risk and use the predetermined cutoff for assigning a risk category. Such risk stratification assists in informing follow-up intensity, guiding triage for pulmonary rehabilitation, while supporting limited resource allocation. In clinical practice, it is necessary to check model performance over time, and update or recalibration is also required for diverse case-mix and care pathways. Enhanced validation, involving prospective impact and temporal validation studies, and data standardization, are necessary for supporting broad model application clinically.

There are certain strengths in the current work. First, model development was completed with data from one institution and assessed with data in another cohort from a second institution. To evaluate the possible overfitting, we utilized 500 bootstrap resamples with optimism correction. Second, cross-validated OOF predictions were used for calibration in the development cohort, with the predetermined operating threshold utilized in the external cohort consistently to reduce *post hoc* tuning and improve reproducibility. Third, we limited predictors to routine pre-discharge data in index admission, and combined laboratory tests and echocardiographic findings. Fourth, SHAP analyses and nomogram development were performed for supporting interpretation and bedside application in personal risk prediction.

Nevertheless, certain limitations should also be acknowledged. Selection bias and unmeasured confounding cannot be avoided considering the retrospective nature of this work. Despite the implementation of external validation, the small external cohort and the low event number would compromise the performance estimate accuracy. Predictors were mainly obtained on the basis of early, single measurements, which cannot indicate post-discharge changes. Post-discharge death can be the competing outcome, however, 90-day mortality was inconsistently recorded among institutions. This makes it impossible to conduct competing risk analysis and may overestimate the readmission probability in patients with an increased short-term mortality risk. Outcome ascertainment also causes a concern: we identified readmissions primarily via the electronic medical record and inpatient information system in each institution, which mainly captured returns to the index hospital. Without insurance claims linkage, regional health information exchange, and standardized post-discharge follow-up, it is impossible to reliably capture admissions to additional hospitals, and crude 90-day COPD/PHD-related readmission rates may be underestimated. In the case of similar missed readmissions among patient groups, the impact was primarily conserved; however, differential capture across institutions cannot be ruled out. This variation, alongside the different catchment populations and access to care, is associated with the different crude readmission rates observed across institutions. More studies that link claims data or regional information platforms and/or implement standardized follow-up will enhance outcome capture and help robustly assess model generalizability. Additionally, different local testing practices and care pathways can also influence model performance.

Collectively, we constructed a prediction model for 90-day readmission and conducted external validation with routine data obtained in index admission, both early in hospitalization and before discharge. LR was identified with the highest stability in the external cohort and shows high interpretability. The model, displayed as a calibrated nomogram, helps identify high-risk patients and instruct post-discharge care planning. Large multi-center cohorts and prospective studies should be performed to verify model performance and optimize the tool prior to clinical translation.

## Data Availability

The raw data supporting the conclusions of this article are currently under further analysis and are available from the corresponding author upon reasonable request.
