# Supplementary material for: Development and external validation of a prediction model for 90-day readmission in elderly patients with COPD complicated by pulmonary heart disease
Source: Front Med (Lausanne). 2026 Jun 11;13:1830474. doi: 10.3389/fmed.2026.1830474 (PMC13295607; doi:10.3389/fmed.2026.1830474)
Supplement: Supplementary file 1 [file Table_1.docx]

## Supplementary Table S1. Multivariable logistic regression coefficients for the final prediction model of 90-day COPD/PHD-related readmission

| **Variables** | **β** | **OR (95% CI)** | **P value** |
| --- | --- | --- | --- |
| ALB（g/L） | -0.0734 | 0.929 (0.865, 0.996) | 0.041 |
| APTT（s） | -0.0446 | 0.956 (0.917, 0.994) | 0.031 |
| EF（%） | -0.0348 | 0.966 (0.930, 1.004) | 0.072 |
| FE |  |  |  |
| No |  | Reference |  |
| Yes | 1.6796 | 5.363 (2.886, 10.280) | <0.001 |
| Gender |  |  |  |
| Female |  | Reference |  |
| Male | 0.4937 | 1.638 (0.805, 3.464) | 0.183 |
| Lymphocyte（10E9/L） | -0.4463 | 0.640 (0.373, 1.006) | 0.072 |
| MPV（fl） | 0.1323 | 1.142 (0.954, 1.372) | 0.151 |
| Monocyte（10E9/L） | 1.2449 | 3.472 (1.251, 9.699) | 0.016 |
| NIV |  |  |  |
| No |  | Reference |  |
| Yes | 0.6767 | 1.967 (1.045, 3.811) | 0.040 |
| TRVmax（m/s） | 2.3560 | 10.549 (5.827, 20.361) | <0.001 |

FE, frequent exacerbations (≥2 hospitalizations for acute exacerbations in the previous year);NIV,non-invasive ventilation;APTT, activated partial thromboplastin time; ALB, albumin; MPV, mean platelet volume; EF, ejection fraction; TRVmax, maximum tricuspid regurgitation velocity;
