# Supplementary material for: Development and external validation of a prediction model for 90-day readmission in elderly patients with COPD complicated by pulmonary heart disease
Source: Front Med (Lausanne). 2026 Jun 11;13:1830474. doi: 10.3389/fmed.2026.1830474 (PMC13295607; doi:10.3389/fmed.2026.1830474)
Supplement: Supplementary file 2 [file Table_2.docx]

**Supplementary Table S2 Calibration and operating threshold**

| **Item** | **Value** |
| --- | --- |
| Platt calibration intercept (a) | -0.084344 |
| Platt calibration slope (b) | 0.886800 |
| Operating threshold | 0.1857 |
| Intercept (β0) | -6.888575 |

Footnote: The raw logistic regression model is logit(p_raw) = β0 + Σ(βi·xi), with β0 = −6.888575 and βi as listed in Table S1 (expanded form: 0.493661×GenderMale + 0.676661×NIVYes + 1.679599×FEYes − 0.044565×APTT − 0.073441×ALB + 0.132345×MPV − 0.446330×Lymphocyte + 1.244862×Monocyte − 0.034774×EF + 2.356036×TRVmax). Probabilities were recalibrated using Platt scaling: p_cal = logistic(a + b·logit(p_raw)), where a = −0.084344 and b = 0.886800. The operating threshold was 0.1857; COPD/PHD-related readmission was predicted when p_cal ≥ 0.1857.
