# Supplementary material for: Development and external validation of a prediction model for 90-day readmission in elderly patients with COPD complicated by pulmonary heart disease
Source: Front Med (Lausanne). 2026 Jun 11;13:1830474. doi: 10.3389/fmed.2026.1830474 (PMC13295607; doi:10.3389/fmed.2026.1830474)
Supplement: Supplementary file 3 [file Table_3.docx]

**Table S3 Baseline characteristics comparison between non-readmission and readmission patients in the main-center cohort.**

| **characteristics** |  | **Non-readmission N=344** | **Readmission N=89** | **P value** |
| --- | --- | --- | --- | --- |
| **Age （year）** |  | 76.1 (6.8) | 76.9 (6.7) | 0.344 |
| **Gender** |  |  |  | 0.089 |
|  | **Female** | 116 (33.7%) | 21 (23.6%) |  |
|  | **Male** | 228 (66.3%) | 68 (76.4%) |  |
| **BMI （kg/㎡）** |  | 21.3 (3.5) | 21.5 (3.7) | 0.676 |
| **Smoke** |  |  |  | 0.550 |
|  | **No** | 167 (48.5%) | 47 (52.8%) |  |
|  | **Yes** | 177 (51.5%) | 42 (47.2%) |  |
| **NIV** |  |  |  | 0.617 |
|  | **No** | 140 (40.7%) | 33 (37.1%) |  |
|  | **Yes** | 204 (59.3%) | 56 (62.9%) |  |
| **RF** |  |  |  | 1.000 |
|  | **No** | 160 (46.5%) | 41 (46.1%) |  |
|  | **Yes** | 184 (53.5%) | 48 (53.9%) |  |
| **FE** |  |  |  | <0.001 |
|  | **No** | 252 (73.3%) | 26 (29.2%) |  |
|  | **Yes** | 92 (26.7%) | 63 (70.8%) |  |
| **Hypertension** |  |  |  | 0.161 |
|  | **No** | 238 (69.2%) | 54 (60.7%) |  |
|  | **Yes** | 106 (30.8%) | 35 (39.3%) |  |
| **CVD** |  |  |  | 0.692 |
|  | **No** | 169 (49.1%) | 41 (46.1%) |  |
|  | **Yes** | 175 (50.9%) | 48 (53.9%) |  |
| **Diabetes** |  |  |  | 0.081 |
|  | **No** | 292 (84.9%) | 68 (76.4%) |  |
|  | **Yes** | 52 (15.1%) | 21 (23.6%) |  |
| **PaO2 （mmHg）** |  | 84.6 (27.9) | 86.9 (34.2) | 0.563 |
| **PaCO2 （mmHg）** |  | 52.7 (17.5) | 51.3 (16.6) | 0.462 |
| **D （Ug/ml）** |  | 2.8 (4.7) | 2.3 (3.6) | 0.237 |
| **TT （s）** |  | 18.4 (1.3) | 18.2 (1.4) | 0.369 |
| **APTT （s）** |  | 37.5 (9.6) | 34.1 (7.9) | <0.001 |
| **PTA （％）** |  | 81.7 (25.5) | 81.9 (23.0) | 0.923 |
| **BNP （pg/ml）** |  | 3154.2 (5161.2) | 3242.1 (5773.3) | 0.896 |
| **ALB （g/L）** |  | 37.6 (4.0) | 36.1 (5.3) | 0.010 |
| **Creatinine （umol/L）** |  | 78.6 (36.1) | 79.3 (30.9) | 0.851 |
| **WBC （10E9/L）** |  | 7.9 (3.7) | 7.8 (3.3) | 0.893 |
| **RBC （10E9/L）** |  | 4.4 (0.8) | 4.3 (0.9) | 0.750 |
| **Hb (g/L)** |  | 129.0 (24.5) | 130.4 (28.1) | 0.681 |
| **HCT （%）** |  | 0.4 (0.1) | 0.4 (0.1) | 0.980 |
| **RDW （%）** |  | 14.8 (2.0) | 14.8 (1.8) | 0.767 |
| **PLT （10E9/L）** |  | 181.0 (79.5) | 198.1 (90.9) | 0.107 |
| **Neutrophil （10E9/L）** |  | 6.3 (3.4) | 6.4 (3.1) | 0.777 |
| **MPV （fl）** |  | 10.5 (1.4) | 10.7 (2.2) | 0.260 |
| **Lymphocyte （10E9/L）** |  | 1.0 (0.6) | 1.0 (0.7) | 0.768 |
| **Monocyte （10E9/L）** |  | 0.5 (0.3) | 0.5 (0.4) | 0.414 |
| **Eosinophils （10E9/L）** |  | 0.1 (0.1) | 0.1 (0.1) | 0.457 |
| **Basophil （10E9/L）** |  | 0.0 (0.0) | 0.0 (0.0) | 0.237 |
| **RVEDD （mm）** |  | 26.4 (5.1) | 26.7 (5.1) | 0.669 |
| **RALD （mm）** |  | 47.6 (8.6) | 46.2 (7.6) | 0.142 |
| **MPAD （mm）** |  | 25.4 (4.4) | 24.9 (4.6) | 0.368 |
| **EF （%）** |  | 62.1 (8.3) | 58.6 (7.6) | <0.001 |
| **TRVmax（m/s）** |  | 3.4 (0.5) | 4.1 (0.5) | <0.001 |
| **PASP （mmHg）** |  | 53.3 (13.1) | 66.8 (14.4) | <0.001 |
| FE, frequent exacerbations (≥2 hospitalizations for acute exacerbations in the previous year);NIV,non-invasive ventilation;RF,Respiratory failure;CVD,Cardiovascular disease(s);D, D-dimer; TT, thrombin time; APTT, activated partial thromboplastin time; PTA, prothrombin activity; BNP, B-type natriuretic peptide; ALB, albumin; WBC, white blood cell count; RBC, red blood cell count; HCT, hematocrit; RDW, red cell distribution width; PLT, platelet count;MPV, mean platelet volume; RVEDD, right ventricular end-diastolic diameter; RALD, right atrial longitudinal diameter; MPAD, main pulmonary artery diameter; EF, ejection fraction; TRVmax, maximum tricuspid regurgitation velocity; PASP, pulmonary artery systolic pressure. | | | | |
